# Evaluation of Probiotic Properties and Safety of *Enterococcus faecium* Isolated From Artisanal Tunisian Meat “Dried Ossban”

**DOI:** 10.3389/fmicb.2018.01685

**Published:** 2018-08-06

**Authors:** Mohamed Zommiti, Mélyssa Cambronel, Olivier Maillot, Magalie Barreau, Khaled Sebei, Marc Feuilloley, Mounir Ferchichi, Nathalie Connil

**Affiliations:** ^1^Unité de Protéomique Fonctionnelle et Potentiel Nutraceutique de la Biodiversité de Tunisie, Institut Supérieur des Sciences Biologiques Appliquées de Tunis, Université de Tunis El Manar, Tunis, Tunisia; ^2^Laboratoire de Microbiologie Signaux et Microenvironnement, Université de Rouen Normandie, Évreux, France; ^3^Clinical Laboratory Department, College of Applied Medical Sciences, King Faisal University, Al-Ahsa, Saudi Arabia

**Keywords:** *Enterococcus faecium*, probiotics, Dried Ossban, safety, antibiotic resistance, virulence determinants

## Abstract

*Enterococcus faecium* strains were isolated from an original biotope, artisanal dried Tunisian meat “Dried Ossban,” and evaluated for safety and capacity as probiotics. Gram-positive, catalase negative, and bacteriocin-producing bacteria were screened using selective microbiological media. All isolates were identified by phenotypic and molecular tools. Five *E. faecium* strains (MZF1, MZF2, MZF3, MZF4, and MZF5) were selected and further assessed for their probiotic properties. They were found to be resistant to the physiological concentrations of bile salts, and the harsh conditions of the gastrointestinal tract, and showed autoaggregation and adhesion ability. All these isolates possess at least one enterocin and could efficiently inhibit the growth of *Listeria innocua* HPB13. The analysis of their safety profile revealed for almost all the strains the absence of cytotoxicity and virulence determinants, and susceptibility to clinically important antibiotics such as vancomycin. These data suggest that these bacteria, isolated from “Dried Ossban,” do not present a risk to human health, and may be considered as interesting candidates for future use as probiotics and bioprotective cultures for application in the food and/or feed industries.

## Introduction

Enterococci are ubiquitous microorganisms, widespread in the environment. In fact, they represent the common members of the commensal microbiota in the intestine of humans, mammals, and other animals encompassing reptiles, birds, and insects, but they are also found in other complex ecosystems such as soil, plants, water, waste, food, and feed (Foulquié Moreno et al., 2006; Byappanahalli et al., 2012). Moreover, *Enterococcus* species are widely present in traditional fermented foods (Giraffa, 2003) due to their amazing capacity to withstand extreme temperature (Murray, 1990), high salinity, and pH levels (Franz et al., 2011). They can grow in the presence of 40% (w/v) bile salts (Fisher and Phillips, 2009). These special characteristics of adaptability to various matrices and conditions may also allow these bacteria to cause spoilage of a wide range of food products, particularly, meat and its derivatives (Björkroth et al., 2005).

Enterococci are increasingly investigated as potential probiotic candidates. Currently, two strains belonging to the genus *Enterococcus* are identified as probiotics and are available on the market, also known as *Enterococcus faecium* SF68^®^ (NCIMB 10415, Cerbios-Pharma SA, Barbengo, Switzerland) and *Enterococcus faecalis* Symbioflor 1 (SymbioPharm, Herborn, Germany). Bybee et al. (2011) have demonstrated the probiotic potential of *E. faecium* SF68^®^ to prevent and treat diarrhea in pets, and *E. faecalis* Symbioflor 1 has been proved efficient for patients suffering from chronic sinusitis and/or bronchitis (Franz et al., 2011). The assessment of the effectiveness of probiotic strains such as *E. faecium* SF68^®^ (NCIMB 10415) and *E. faecalis* Symbioflor 1 in humans for the treatment of diarrhea, irritable bowel syndrome, in lowering serum cholesterol and for immune regulation has been investigated in very few studies (Franz et al., 2011; Christoffersen et al., 2012; Yamaguchi et al., 2013). As a result, there are not enough data to firmly conclude on the efficiency of enterococci as probiotics for humans and their use in foods must be based on case-by-case investigations (Ogier and Serror, 2008).

Producing antimicrobial substances preventing pathogens can be a reliable criterion when selecting probiotic strains. Among lactic acid bacteria (LAB), enterococci are known to secrete various antimicrobial compounds, so these bacteria seem potentially useful to prevent diseases of bacterial foodborne origins (Franz et al., 2011). Numerous enterococci produce at least one bacteriocin, a bioactive substance of proteinaceous nature, which is ribosomally synthesized and active against a broad collection of spoiling and foodborne microbes encompassing *Listeria* spp. (Favaro et al., 2014). In the last decade, numerous studies have been published on bacteriocin-producing enterococci in association with food ecosystems (Todorov et al., 2010; Hadji-Sfaxi et al., 2011) and their isolation from various ecological biotopes, encompassing meat (Cintas et al., 1995), vegetables (Bennik et al., 1998), soil (Yanagida et al., 2005), crops (Todorov et al., 2005), fish (Satish Kumar et al., 2011), dairy products (Chanos and Williams, 2011), and humans (Gálvez et al., 1986).

However, Enterococci have also gained notoriety and sturdiness over the past few years as the leading nosocomial pathogens (Rosenthal et al., 2008). According to Schaberg et al. (1991), enterococcal species are well recognized as the second-most common causal agent of urinary tract infections and the third-most for nosocomial bacteraemia. Clinically, vancomycin-resistant Enterococci (VRE) represent a major problem in nosocomial infections (Arias and Murray, 2012). These bacteria often possess multiple antibiotic resistances and virulence factors such as hemolysin, adhesins, and invasins (Ogier and Serror, 2008; Franz et al., 2011). For all the reasons previously mentioned, enterococci species are generally considered to have a doubtful status for food safety (Ladero et al., 2012).

In this work, we report for the first time the isolation and characterization of five enterococci from “Dried Ossban,” a traditional Tunisian fermented meat product. Their safety, probiotic potential, and antimicrobial properties have been investigated.

## Materials and Methods

### Sampling and Isolation of Lactic Acid Bacteria (LAB)

Lactic acid bacteria strains were isolated from “Dried Ossban,” a Tunisian traditional dry fermented meat typically prepared from sheep intestine and meat mixed with salt and spices and dried through exposure for several days to sunlight. The samples were obtained from homemade production of fermented meat, from various governorates covering almost all the Tunisian territory. Samples were collected into sterile bottles and then transported on ice to the laboratory for analyses. For all samples, 10 g was added to 90 ml of sterile peptone saline water and homogenized for 10 min through vortexing at maximum speed. Appropriate decimal dilutions were plated onto de Man Rogosa Sharpe (MRS) agar and incubated at 37°C for 24–48 h. Colonies were randomly selected from MRS agar, and only Gram-positive and catalase-negative isolates were retained, routinely propagated, and stored at -80°C in MRS broth containing 20% glycerol. For experimental assays, working cultures were sub-cultured (1% inoculum, 24 h, 37°C) prior to use.

### Identification of the LAB Isolates by Matrix-Assisted Laser Desorption Ionization-Time-of-Flight Mass Spectrometry (MALDI-TOF MS)

The Gram-positive and catalase negative LAB isolated from “Dried Ossban” were identified by analysis of the total proteome using an Autoflex III matrix-assisted laser desorption/ionization-time-of-flight mass spectrometer (MALDI-TOF MS; Bruker, Marcy-l’Etoile, France) coupled to the MALDI-Biotyper 3.1 algorithmic system for microbial identification. Briefly, each bacterium was cultured on MRS agar for 24 h and the colonies were analyzed by direct spot on the MALDI target plate or using the 70% formic acid protein extraction method, as previously described (Hillion et al., 2013).

The MALDI target plate was introduced into the MALDI-TOF MS for automated measurement and data interpretation. The instrument was calibrated using a Bruker bacterial test standard and spectra were acquired in the linear mode over a mass range from 2000 to 20,000 Da. The spectrum of each unknown bacterium was electronically transformed into the peak list and compared to the 6903 reference organisms in the database and a log(score) value between 0.00 and 3.00 was generated which represents the probability that the match is correct. A score of ≥1.7 indicates genus identification, and a score of ≥2.0 is the set threshold for determination at the species level (Sogawa et al., 2011).

### Acid and Bile Salt Tolerance

For the acid tolerance assays, MRS broth was used to simulate the acidity of the gastrointestinal tract after adjusting to pH 3.0 with 1 N HCl. Fresh overnight cultures of *E. faecium* strains isolated from “Dried Ossban” and previously identified by MALDI-TOF MS were centrifuged (8000 rpm, 10 min), washed with phosphate buffer saline (PBS), and resuspended to approximately 10^7^ bacteria/ml in the MRS broth pH 3.0. Samples (0.1 ml) were taken at 0 h and after 1, 2, and 3 h incubation at 37°C, simulating the time spent in the human stomach. The number of bacterial cells was then enumerated by the pour plate method of all samples using decimal serial dilutions prepared in PBS, and after 24 h incubation at 37°C. Survival bacteria were expressed as log values of colony-forming units per ml (CFU/ml).

The bile salt tolerance was tested as previously described by Anandharaj et al. (2015) with slight modifications. Bacteria from overnight cultures were inoculated (1% v/v) into MRS broth containing 0.3% (w/v) Ox-bile (Sigma–Aldrich). Total viable counts were determined after 0, 1, 2, and 3 h of exposure to bile salts, reflecting the time that food spends in the small intestine. Aliquots were diluted, plated on MRS agar, and incubated at 37°C for 24 h. Samples without addition of bile salts served as controls. Survival bacteria were expressed as described above for acid tolerance.

### Autoaggregation

Autoaggregation of the *E. faecium* strains isolated from “Dried Ossban” was assessed according to Collado et al. (2008). Overnight cultures were centrifuged (8000 rpm, 10 min), washed twice with PBS, and resuspended in the same buffer to approximately 10^8^ bacteria/ml. Each suspension (4 ml) was vortexed for 20 s and incubated at room temperature. The absorbance at 600 nm (A_600_) of the upper part of each suspension was measured at time 0 h and 24 h after incubation without vortexing. The percentage of autoaggregation of each strain was then calculated according to the following equation:

Autoaggregation (%) = [1 - (*A*_Time_/*A*_0_) × 100], where *A*_Time_ refers to the absorbance of the suspension at 24 h and *A*_0_ refers to the absorbance at time 0.

### Caco-2/TC7 Culture

The human colon adenocarcinoma cell line Caco-2/TC7 (passages 40–60) were routinely grown in Dulbecco’s Modified Eagle’s Medium (DMEM, Invitrogen, France), supplemented with 15% heat-inactivated fetal calf serum (FCS), and 100 U/ml each of penicillin and streptomycin. The cells were cultivated at 37°C in 5% CO_2_–95% air atmosphere, and the medium was regularly changed. For adhesion and cytotoxicity assays, the cells were seeded in 24-well tissue culture plates and incubated to confluence, and for transepithelial electrical resistance (TEER) measurements, they were grown on inserts (3 μm pore size) until full differentiation (21 days).

### *In vitro* Adhesion Assays

*E. faecium* strains, grown overnight in MRS broth, were harvested by centrifugation (8 000 rpm, 10 min), washed twice in PBS solution, and resuspended in cell culture medium, without serum and antibiotic, at a concentration of 10^8^ bacteria/ml, and then applied on confluent Caco-2/TC7 monolayers as previously described for other bacteria (Messaoudi et al., 2012). Briefly, after 3 h of incubation at 37°C, in 5% CO_2_–95% air atmosphere, monolayers were gently washed three times with sterile pre-warmed PBS, to remove non-adherent bacteria, and disrupted by incubation for 15 min with 0.1% Triton X100. The lysates were then diluted and plated onto MRS agar to determine the number of adherent bacteria.

### Cytotoxicity Assay

The cytotoxicity was determined using an enzymatic assay (Cytotox 96 Promega, France) which measures lactate dehydrogenase (LDH) released from the cytosol of damaged Caco-2/TC7 cells into the supernatant. LDH is a stable cytosolic enzyme present in many different cell types, particularly in eukaryotic cells, playing the role of an indicator of necrotic cell death when released. After overnight incubation with the *E. faecium* strains (10^8^ bacteria/ml), the supernatants from confluent Caco-2/TC7 monolayers grown on 24-well tissue culture plates were collected and the concentration of the LDH was quantified. Two controls were included for calculation of cytotoxicity percentage. Caco-2/TC7 cells exposed to Triton X100 (0.9%) were used as a positive control of maximal LDH release (100% lysis) as specified by the manufacturer’s recommendations. The background level (0% LDH release) was determined with serum-free culture medium.

### Transepithelial Electrical Resistance (TEER)

The TEER of differentiated Caco-2/TC7 cells was monitored during 24 h using the Millicell Electrical Resistance System (Millipore Corp., Bedford, MA, United States). To study the potential effect of *E. faecium* strains on the epithelial barrier integrity, these bacteria were incubated at 10^8^ bacteria/ml on the Caco-2/TC7 cell monolayers. Control monolayers were not exposed to the potential probiotics. TEER values were expressed as percentages of the initial level measured in the insert.

### DNA Extraction

Bacterial cells from overnight cultures in MRS broth of the five *E. faecium* strains identified from “Dried Ossban” were harvested by centrifugation at 8000 rpm for 10 min. Total genomic DNA was extracted and highly purified via GeneJET Genomic DNA Purification Kit (Thermo Scientific, France) according to the manufacturer’s recommendations. DNA quality and concentration were determined by measuring optical density (OD) at 260 and 280 nm with a spectrophotometer.

### Screening for Genes Encoding Virulence Determinants and Resistance to Vancomycin

The virulence factors of the *E. faecium* strains were investigated by PCR amplification, revealing the presence of genes encoding for aggregation substance (*agg*), gelatinase (*gelE*), enterococcal surface protein (*esp*), and collagen adhesin (*ace*), (Eaton and Gasson, 2001; Reviriego et al., 2005). Primer sequences and fragment sizes are presented in **Table 1**. PCR for virulence determinant genes was performed using PCR protocols of (Mannu et al., 2003; Reviriego et al., 2005). The amplified products were separated by electrophoresis on 1% (w/v) agarose gels in 1× Tris-acetate-EDTA (TAE) buffer. Gels were stained with SYBR Safe DNA gel stain and visualized under UV light.

**Table 1:** PCR primers used for detection of virulence determinants, antibiotic resistance, biogenic amines, and enterocin genes.

Target genes	Oligonucleotide sequence (5′–3′)	Product size (bp)	Reference
**Virulence**			
*agg* (aggregation substance)	F: AAGAAAAAGAAGTAGACCAAC	1553	Eaton and Gasson, 2001
	R: AAACGGCAAGACAAGTAAATA		
*gelE* (gelatinase)	F: ACCCCGTATCATTGGTTT	419	
	R: ACGCATTGCTTTTCCATC		
*esp* (enterococcal surface protein)	F: TTGCTAATGCTAGTCCACGACC	933	
	R: GCGTCAACACTTGCATTGCCGAA		
*ace* (adhesion of collagen protein)	F: AAAGTAGAATTAGATCACAC	350	Duprè et al., 2003
	R: TCTATCACATTCGGTTGCG		
**Antibiotic resistance**			
*vanA* (vancomycin resistance)	F: CCCCTTTAACGCTAATACGATCAA	1030	Clark et al., 1993
	R: CATGAATAGAATAAAAGTTGCAAT		
*vanB* (vancomycin resistance)	F: GTGACAAACCGGAGGCGAGGA	433	
	R: CCGCCATCCTCCTGCAAAAAA		
**Biogenic amines**			
*hdc1* (histidine decarboxylase)	F: AGATGGTATTGTTTCTTATG	367	Khalkhali and Mojgani, 2017
	R: AGACCATACACCATAACCTT		
*hdc2* (histidine decarboxylase)	F: AAYTCNTTYGAYTTYGARAARGARG	534	
	R: ATNGGNGANCCDATCATYTTRTGNCC		
*tdc* (tyrosine decarboxylase)	F: ACATAGTCAACCATRTTGAA	1100	
	R: CAAATGGAAGAAGAAGTAGG		
**Enterocins**			
*Ent A* (*Enterocin* A)	F: AAATATTATGGAAATGGAGTGTAT	126	Du Toit et al., 2000
	R: GCACTTCCCTGGAATTGCTC		
*Ent B* (*Enterocin* B)	F: GAAAATGATCACAGAATGCCTA		
	R: GTTGCATTTAGAGTATACATTTG	162	
*Ent P* (*Enterocin* P)	F: TATGGTAATGGTGTTTATTGTAAT		Wieckowicz et al., 2011
	R: ATGTCCCATACCTGCCAAAC	112	


The strains were also evaluated for *vanA* and *vanB* genes (both related to vancomycin resistance) by PCR reaction according to the protocol of Clark et al. (1993). The primer sequences are described in **Table 1**.

### Detection of Amino Acid Decarboxylase Genes

The potential of the *E. faecium* strains to produce histamine and tyramine was assessed via molecular tools. Genes related to the production of these biogenic amines are part of the genetic heritage of *Enterococcus* spp. (Connil et al., 2002). Analysis of the histidine and tyrosine decarboxylase encoding genes (*hdc* and *tdc*, respectively) in MZF1–MZF5 strains was carried out by PCR amplification with the primers previously used by Khalkhali and Mojgani (2017). PCR primers and sizes of the resulted amplicons are listed in **Table 1**. PCR reactions were performed using the following cycling parameters: initial denaturation for 5 min at 95°C followed by 35 cycles of 1 min at 95°C, annealing at 54°C for 30 s, and 1 min at 72°C and a final extension step of 5 min at 72°C.

### Detection of Bacteriocin Structural Genes by PCR and Antibacterial Activity

The presence of the main enterocin genes was screened by PCR amplification. Total DNA from the five *E. faecium* strains was submitted to PCR reactions to detect genes responsible for codification of the following bacteriocins: enterocin A, enterocin B, and enterocin P (**Table 1**). The PCR amplification conditions were as already described (Du Toit et al., 2000; Wieckowicz et al., 2011).

For detection of antibacterial activity, a well agar diffusion test of the *E. faecium* isolates was carried out as previously (Jacobsen et al., 1999) using smooth brain heart infusion (BHI) agar, containing the indicator strains (*Listeria innocua* HPB13, *E. faecalis* ATCC 29212, *Staphylococcus aureus* ATCC 25923, or *Escherichia coli* DH5α).

### Antibiotic Susceptibility Testing

The antibiotic susceptibility of the *E. faecium* strains was checked by the broth microdilutions method. The antibiotics tested were ampicillin, vancomycin, gentamycin, erythromycin, ofloxacin, tetracycline, and chloramphenicol (**Table 2**). In brief, 100 μl of *E. faecium* strains (1.10^5^ bacteria/well final concentration) were inoculated into the wells of a 96-well plate containing 100 μl of each antibiotic in serial twofold dilutions from 512 to 0.125 μg/ml, and then were incubated for 24 h. The results were compared with growth control (enterococci alone), and the minimum inhibitory concentration (MIC) breakpoint is the concentration where no growth is observed. The level of susceptibility to antibiotics was reported as sensitive, intermediate or resistant according to the observed breaking points recommended for enterococci by the Clinical and Laboratory Standards Institute (CLSI) antimicrobial susceptibility testing standards (CLSI, 2015).

**Table 2:** List of antibiotics used in the study.

Name of drug	Antibiotic group	Mode of action
Ampicillin	β-Lactams	Inhibitors of the cell wall synthesis
Vancomycin	Glycopeptides	
Ofloxacin	Quinolones	Inhibiting DNA replication and transcription
Gentamycin	Aminoglycosides	Inhibitors of protein synthesis
Erythromycin	Macrolides	
Chloramphenicol	Others	
Tetracycline	Tetracyclines	


### Statistical Analysis

Data are expressed as a mean ± standard error (SE) calculated over three independent experiments performed in triplicate. Analysis of statistical significance was performed by ANOVA. Student’s *t*-test was used, when necessary, to discriminate differences between means. Differences with *P*-value < 0.05 were considered statistically significant.

## Results and Discussion

### Characterization of *Enterococcus faecium* Strains

**FIGURE 1:**
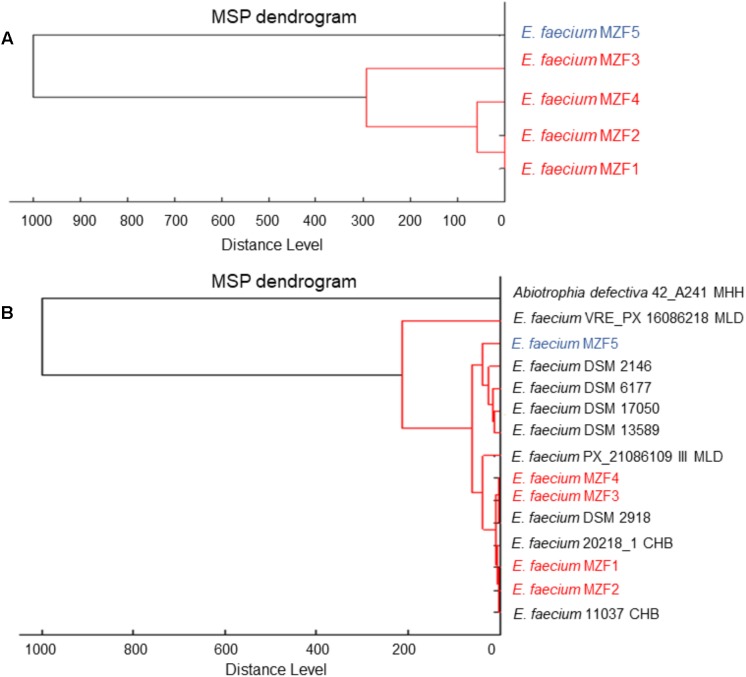
MALDI-TOF MS based phylogenetic tree. **(A)** Distance level between the *E. faecium* strains isolated from Tunisian “Dried Ossban” and **(B)** distance level of the isolates compared to the *E. faecium* reference strains available in the Biotyper library.

Thirty-seven samples of artisanal dried Tunisian meat “Dried Ossban” were collected from homemade production in different governorates of the Tunisian territory to isolate potential probiotics from the lactic acid flora. The strains were first presumptively identified by phenotypic analysis (Gram staining, cellular morphology, catalase, and oxidase tests). Five presumptive *Enterococcus* strains (Gram^+^ coccus, catalase, and oxidase negative) were selected for further study and called MZF1, MZF2, MZF3, MZF4, and MZF5. Their growth in various conditions of temperature, salinity, and pH was examined. All these strains were able to grow at 45°C, in 6.5% NaCl, and at pH 9.6 but not pH 4. These characteristics are well known for *Enterococcus* as this genus has an extraordinary capacity to grow under hostile conditions. MZF1, MZF2, MZF3, MZF4, and MZF5 were then analyzed by Maldi Biotyper for confirmation of the genus *Enterococcus* and determination of the species. The five strains were identified as *E. faecium* with score > 2 and accordingly to the library of Maldi Biotyper, a dendrogram was generated from the minimal spanning tree (MSP) data set (Elbehiry et al., 2017). For this, the main spectra of the Maldi Biotyper taxonomy were compared with the spectra resulting in a matrix of cross-wise identification scores. This matrix was applied to estimate the distance level for each pair of main spectra. A dendrogram was created according to the distance level between the *E. faecium* strains isolated from Tunisian “Dried Ossban” (**Figure 1A**) and with the *E. faecium* reference strains available in the Biotyper library (**Figure 1B**). This phyloproteomic analysis shows the hierarchical relationship between the five isolates and suggests that *E. faecium* MZF5 is more distant from the four other “Dried Ossban” strains.

Simultaneously, a PCR amplification with the *E. faecium* specific primers (EfmF1 5′-ACGGAGATCGTGGATTCAAA-3′ and EfmR1 5′-CGTACGGGAAGTGATTCGAC-3′) from Park et al. (2017) has been done and gave positive results. Indeed, as expected, a PCR product of 1032 bp was obtained, corresponding to the specific amplification of the M protein trans-acting positive regulator target gene of *E. faecium* (data not shown). Finally, 16S rRNA genes of the five *E. faecium* isolates (MZF1, MZF2, MZF3, MZF4 and MZF5) have been sequenced and the partial nucleotide sequences have been deposited in the NCBI Genbank under the accession numbers MH569603, MH569604, MH588167, MH591462, and MH591463, respectively.

### Acid and Bile Salt Tolerance

The efficiency of a bacterium as a potential probiotic depends firmly on its aptitude to survive the passageway through the upper digestive tract to the intestine, where its beneficial effect is expected, and this represents a crucial requirement (Marteau et al., 1997; Tuomola et al., 2001). According to Maragkoudakis et al. (2006), the pH inside the human stomach ranges from 1 (during fasting) to 4.5 (after a meal), and food ingestion can take up to 3 h. Our findings indicated that the five isolates exhibited high tolerance to acidity after exposure to media pH 3 (**Table 3**) and were also tolerant to bile salts, since all of them survived successfully in MRS broth medium supplemented with 30 g/l bile salt, reflecting the physiological concentration of human bile (**Table 4**). Our results are in total accordance with data published by other authors for *Enterococcus* spp. isolated from food niches suggesting that these “Dried Ossban” strains could have the potential to reach the intestinal lumen and thus stay alive in that environment (Ahmadova et al., 2013; Gupta and Tiwari, 2015; Aspri et al., 2016).

**Table 3:** Low pH and acidic conditions tolerance.

Isolates	Mean of viable count (log_10_ CFU/ml)			
	Time of exposure (h)			
	0	1	2	3
MZF1	6.71	6.46	6.50	6.47
MZF2	6.61	6.59	6.52	6.49
MZF3	7.02	6.89	6.61	6.57
MZF4	6.69	6.51	6.43	6.39
MZF5	7.09	6.92	6.79	6.67


**Table 4:** Bile salts tolerance.

Isolates	Mean of viable count (log_10_ CFU/ml)			
	Time of exposure (h)			
	0	1	2	3
MZF1	6.67	6.59	6.54	6.51
MZF2	6.57	6.54	6.49	6.44
MZF3	7.01	6.74	6.62	6.50
MZF4	6.62	6.57	6.51	6.42
MZF5	7.04	6.76	6.59	6.54


### Autoaggregation

**FIGURE 2:**
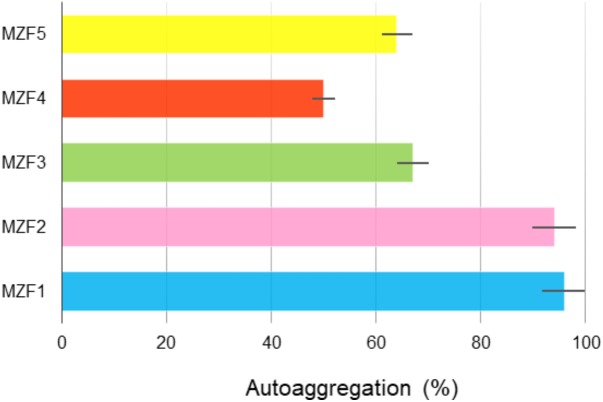
Autoaggregation of the *E. faecium* strains isolated from “Dried Ossban.” Data are expressed as mean ± SE.

According to the investigations of Collado et al. (2007), cell aggregation properties represent one of the most important phenotypic characteristics of a potential probiotic strain. In this study, the ability of the enterococcal strains, isolated from “Dried Ossban,” to aggregate was evaluated. The results showed autoaggregation values ranged from 50 to 96% (**Figure 2**). MZF4 showed the lower level, 50%, whereas MZF2 and MZF1 showed the highest levels with 94 and 96%, respectively. These findings are supported by previous data demonstrating high values of autoaggregation for *E. faecium* strains (Dos Santos et al., 2015; Veljović et al., 2017). Autoaggregation proved to be strain-specific and may vary inside the same taxonomic group (Dos Santos et al., 2015). This strain specificity in autoaggregation has been reported in a study led by Todorov et al. (2008) for *Lactobacillus pentosus* ST712BZ and *Lactobacillus paracasei* ST284BZ. Bacterial autoaggregation is considered an important phenomenon in several ecological niches (Del Re et al., 2000). Among LAB, enterococcal species represent beneficial members of the microbiota residing the human and animal gastrointestinal and urogenital tracts (Bhardwaj et al., 2009). One beneficial feature of bacteria with autoaggregative potential is the ability to prevent the colonization of pathogenic bacteria via the formation of a barrier through autoaggregation to intestinal mucosa (Prince et al., 2012).

### Adhesion Capacity

**FIGURE 3:**
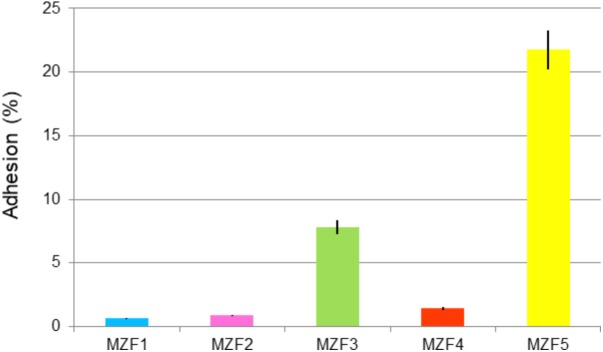
Adhesion of the *E. faecium* isolates to the intestinal Caco-2/TC7 cells. Data are expressed as mean ± SE.

Saarela et al. (2000) demonstrated the importance of the adhesion step. Once the probiotic bacteria have reached the gut, the adhesion to the intestinal mucosa and epithelial cells represents a requirement step for colonization. Probiotics must adhere to the mucus layer to prevent being removed from the colon by peristalsis. To complete the study of the *E. faecium* isolated from “Dried Ossban,” *in vitro* assessment of their adhesive potential to human colon adenocarcinoma epithelial cells (Caco-2/TC7 cell line) was conducted. **Figure 3** shows three isolates with very low adhesion percentages, 0.7, 0.9, and 1.4% for MZF1, MZF2, and MZF4, respectively. A moderate value is noticed with MZF3 isolates, 7.8% of adhesion, and the highest value is accorded to the MZF5 isolate with more than 21% of adhesive potential to Caco-2/TC7 cells. This agrees with other studies demonstrating strong adhesiveness for *E. faecium* BGGO9-28 (Veljović et al., 2017) and other LAB strains than Enterococcal species (Juntunen et al., 2001). Our findings showed a variability of results among the isolates. This is in line with Turpin et al. (2012) who reported that 30 LAB strains displayed adhesion capacities from 0.6 to 30.0% on the HT-29 cells. For the MZF5 isolate with 21.8% of adhesion capacity, this value was greater than that reported for the *E. faecalis* UGRA10 (Cebrián et al., 2012). It is significant to highlight that *in vitro* experiments have many restrictions, since adhesion properties and mucus production are strictly cell line-dependent in every single study (Turpin et al., 2012). *In vitro* test results using Caco-2 cells for the evaluation of adhesion ability to intestinal epithelium cells have been reported to have a good correlation with *in vivo* results (Crociani et al., 1995) and this characteristic is often strain-specific (Duary et al., 2011).

### Cytotoxicity

**FIGURE 4:**
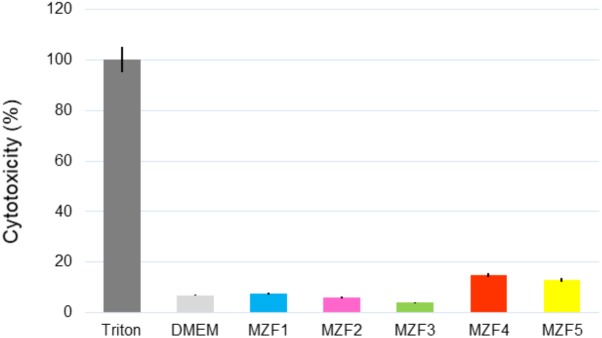
Cytotoxicity of the *E. faecium* isolates measured by LDH release after overnight incubation of Caco-2/TC7 cells with 10^8^ bacteria/ml. Data are expressed as mean ± SE.

As *E. faecium* contains concurrently harmless and pathogenic strains, a cytotoxicity assay was needed to ensure the safety of MZF1, MZF2, MZF3, MZF4, and MZF5. The five strains were applied overnight on Caco-2/TC7 monolayers and the cytotoxicity was estimated by measurement of LDH release and microscopic observation. Our findings showed that the weakest level of cytotoxicity is attributed to the MZF3 isolate with 4% of mortality, reflecting a high rate of viability, about 96%. This isolate exhibited no significant effect on the viability of Caco-2 cells compared to cells alone. MZF1 and MZF2 showed about 8 and 6% of cytotoxicity rate; 15 and 13% of the mortality was obtained with strains MZF4 and MZF5, respectively (**Figure 4**). These results are in harmony with previous studies showing that various probiotic bacteria had no cytotoxic effect on Caco-2 cells. For instance, Er et al. (2015) studied the cytotoxicity of three LAB (*Pediococcus pentosaceus, Lactobacillus plantarum*, and *Weissella confusa*) on Caco-2 cells and showed dose-dependent cytotoxic effects of the cell-free filtrates and cell-free lyophilized filtrates of the three LAB. Similarly, a recent work performed by Castro et al. (2016) demonstrated that *E. faecalis* CECT7121 strain is not cytotoxic for Caco-2 cells.

### Transepithelial Electric Resistance (TEER)

**FIGURE 5:**
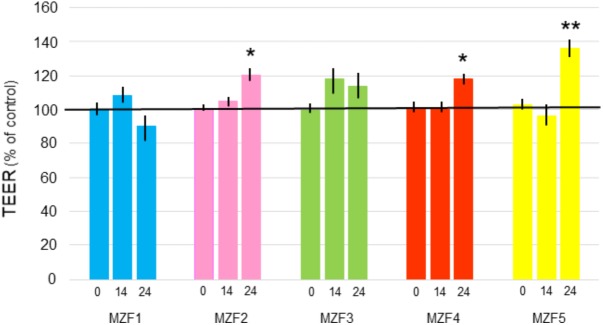
TEER at time 0, 14, and 24 h of Caco-2/TC7 cells exposed to the *E. faecium* isolates (10^8^ bacteria/ml). Data are expressed as percentages of the initial level measured in the insert and as a mean ± SE. ^∗^*P* < 0.05 and ^∗∗^*P* < 0.01 compared to Caco-2/TC7 control.

Measuring the TEER is a method for evaluating the intestinal barrier integrity *in vitro* (Schneeberger and Lynch, 2004). Results found for the effect of “Dried Ossban” isolates on Caco-2/TC7 monolayers are presented in **Figure 5**. After 24 h of incubation, MZF1 and MZF3 showed no significant impact on TEER, whereas MZF2 and MZF4 led to a slight 20% increase of TEER, and MZF5 a 30% increase. These findings are in agreement with the results obtained for other well-known and characterized probiotics [*Bifidobacterium infantis, Lactobacillus acidophilus, Lactobacillus rhamnosus, E. coli* Nissle 1917, *Streptococcus thermophilus*, the probiotic complex VSL#3] on different cell lines (T84, HT29/cl.19A, and Caco-2) (Resta-Lenert and Barrett, 2003; Sherman et al., 2005; Ewaschuk et al., 2008). Indeed, it has been demonstrated that the application of probiotics on intestinal epithelial cells can give rise to two kinds of response. The first one was a significant enhancement of TEER values (Resta-Lenert and Barrett, 2003; Ewaschuk et al., 2008; Klingspor et al., 2015), while the other type of response was no effect on TEER measurements (Sherman et al., 2005). Furthermore, the effects were frequently showed to be dose- and time-dependent (Klingberg et al., 2005).

### Virulence Factors

Virulence determinants greatly contribute to enhancing infection because of the risk of genetic transfer. Since these genes are usually located in conjugative plasmids (Eaton and Gasson, 2001), potential virulence genes of *E. faecium*, isolated from food sources, need to be assessed. Therefore, it seemed needful to test the five *E. faecium* isolates for their virulence factors profile (**Table 5**). Results showed that only the isolate MZF1 carried the *agg* gene, whereas *gelE* gene was detected in MZF1 and MZF4. The MZF2, MZF3, and MZF5 isolates do not harbor any of the tested virulence genes. Our findings are consistent with the data reported by Zheng et al. (2015), proving the absence of these virulence factors in food-isolated *E. faecium* strains, and with the study conducted by Eaton and Gasson (2001) who showed that the presence or absence of virulence factors is strain-specific. Indeed, Eaton and Gasson (2001) and Ahmadova et al. (2013) reported that a high frequency of the *esp* gene is only found in medical *E. faecium* strains. Besides, the presence of some virulence determinants such as *agg* and *gelE* genes in MZF1 and MZF4 cannot be considered as a negative trend since some commercial enterococci starter cultures with a long history of safe use are also known to possess several virulence genes such as *ace, asa1, gelE*, and *esp*. Moreover, Cebrián et al. (2012) and Popović et al. (2018) suggested that some virulence factors as the agg substance and the esp surface protein may play a beneficial role for probiotic bacteria. In fact, this may help the bacterium colonizing the gastrointestinal tract, and proliferating inside it, revealing its probiotic properties.

**Table 5:** PCR results of virulence and antibiotic resistance genes.

*E. faecium* isolates	Genotype profile
MZF1	agg^+^ gelE^+^ esp^-^ ace^-^ vanA^-^ vanB^-^
MZF2	agg^-^ gelE^-^ esp^-^ ace^-^ vanA^-^ vanB^-^
MZF3	agg^-^ gelE^-^ esp^-^ ace^-^ vanA^-^ vanB^-^
MZF4	agg^-^ gelE^+^ esp^-^ ace^-^ vanA^-^ vanB^-^
MZF5	agg^-^ gelE^-^ esp^-^ ace^-^ vanA^-^ vanB^-^


### Histamine and Tyramine Production

**FIGURE 6:**
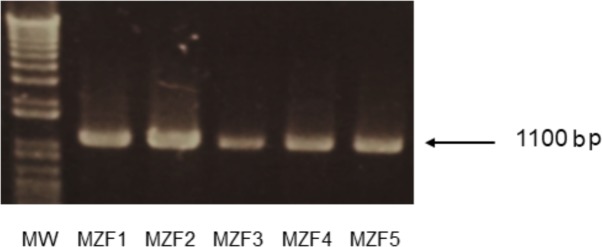
PCR amplification of the tyrosine decarboxylase (*tdc*) gene in the five *E. faecium* strains isolated from “Dried Ossban” Molecular Weight marker (MW) 1 kB.

The isolated DNA of the *E. faecium* strains was subjected to PCR amplification to detect the presence of histidine and tyrosine decarboxylase encoding genes (*hdc* and *tdc*, respectively). All the five strains showed the expected amplicon of 1100 bp corresponding to the *tdc* gene (**Figure 6**) but none harbored the histidine decarboxylase encoding gene, *hdc*. Our findings are in line with studies conducted by Inoǧlu and Tuncer (2013), Ruiz et al. (2016), and Avci and Özden Tuncer (2017) where the authors found that all the analyzed *E. faecium* strains did not harbor the *hdc* gene but had the *tdc* gene. Histamine is the more toxic biogenic amine but the level of tyramine in food must also be controlled. A physiological assay showed that the five “Dried Ossban” strains were not, or weak producers of tyramine (data not shown). This will have to be confirmed later by a direct inoculation test of these bacteria in meat product.

### Antibiotic Susceptibility

Antibiotic resistance represents an essential issue in the safety evaluation of *Enterococcus* spp. Based on the broth microdilution procedure, all enterococci isolates showed sensitivity toward ampicillin and vancomycin (**Table 6**) which represent the commonly used antibiotics to treat enterococcal infections (Favaro et al., 2014). Vancomycin susceptibility was expected as PCR detection of the antibiotic resistance genes vanA and vanB gave negative results (**Table 5**). The five “Dried Ossban” strains were also sensitive to chloramphenicol, gentamicin, and tetracyclin, whereas they showed resistance to erythromycin (Macrolides) and ofloxacin (Quinolones).

**Table 6:** Antibiotic susceptibility.

Antibiotic	MIC breakpoint recommendation of CLSI for *Enterococcus* spp. (μg/ml)			Strain/antibiotic susceptibility				
	S	I	R	MZF1	MZF2	MZF3	MZF4	MZF5
Ampicillin	≤8	–	≥16	<1 (S)	0.25 (S)	<0.125 (S)	<0.125 (S)	<0.125 (S)
Vancomycin	≤4	8–16	≥32	2 (S)	2 (S)	2 (S)	2 (S)	2 (S)
Ofloxacin	≤1	2	≥4	32(R)	32 (R)	16 (R)	32 (R)	32 (R)
Gentamicin	≤500	N/A	≥500	128 (S)	128 (S)	256 (S)	256 (S)	256 (S)
Erythromycin	≤0.5	1–4	≥8	8 (R)	8 (R)	4 (I)	16 (R)	16 (R)
Chloramphenicol	≤8	16	≥32	2 (S)	8 (S)	4 (S)	4 (S)	4 (S)
Tetracyclin	≤4	8	≥16	<1(S)	<1(S)	<1 (S)	<1(S)	<1 (S)


*CLSI, Clinical and Laboratory Standards Institute; MIC, minimal inhibitory concentration; N/A, not applicable; R, resistant; S, susceptible.*

The major criterion for the safety assessment of enterococci is their susceptibility to glycopeptides such as vancomycin. Fortunately, Franz et al. (2001) reported that vanA and vanB genes are rarely detected in enterococci isolates from food sources, which is in congruence with our results. Moreover, whereas many strains of *Enterococcus* are known to be intrinsically resistant to β-lactam antibiotics (de Fátima Silva Lopes et al., 2005), the five *E. faecium* isolated from “Dried Ossban” were sensitive to ampicillin. Concerning aminoglycosides, high rates of resistance have been previously noticed in enterococcal strains from dairy products (Hammad et al., 2015; Aspri et al., 2016). On the contrary, all the “Dried Ossban” isolates were sensitive to gentamicin. High levels of tetracycline resistance have been found previously in Enterococci from animal origin, and this may be due to the wide use of this antibiotic in husbandry activities (Busani et al., 2004). It is worth noting that on the contrary, the strains isolated from “Dried Ossban” were all sensitive to tetracycline. These bacteria were also sensitive to chloramphenicol, and this is in harmony with previous studies (Zheng et al., 2015; Aspri et al., 2016). Conversely, a high rate of erythromycin resistance was detected among the “Dried Ossban” Enterococci, 80% (4/5 isolates) and the remaining 20% (1/5 isolates) were classified as intermediate. Macrolides are frequently used in animal husbandry; this could contribute to the emergence of many resistant strains (Diarra et al., 2010).

For safety reasons, a critical criterion for *Enterococcus* to be used in foods is the lack of transferable antibiotic resistance (Cebrián et al., 2012). All the isolates in this study showed sensitive traits to five antibiotics.

### Detection of Bacteriocin Structural Genes by PCR

Numerous studies have reported the potential of several enterococcal species such as *Enterococcus mundtii, E. faecium*, and *E. faecalis* to produce bacteriocins, also known as enterocins, highly active against sturdy spoilage and pathogenic microorganisms in foods and food products, such as *Listeria* spp. (Favaro et al., 2014). Most of these enterocins have been genetically characterized; it seems that most of them are class II bacteriocins, heat stable, cationic, hydrophobic, and low molecular weight peptides. The classification of enterocins includes four main classes. Class II is subdivided into three subclasses: the pediocin family of enterocins such as enterocin A and enterocin P (IIa) with strong anti-listerial effects, bacteriocins synthesized without a leader peptide such as enterocin L50 A and B (IIb), linear enterocins that do not belong to the pediocin family (IIc) and the class (IId) including enterocin B (Aymerich et al., 1996; Casaus et al., 1997; Franz et al., 2007). Bacteriocin production is being increasingly considered as an important criterion in the selection of a probiotic strain (Dobson et al., 2012; Hegarty et al., 2016). In our study, MZF1, MZF2, MZF4, and MZF5 have been shown to be highly active against *L. innocua* HPB13 (**Table 7**). These isolates were found to harbor the *EntA, EntB*, and *EntP* genes, whereas the MZF3 isolate has been shown carrying out only the *EntB* gene with a weak activity against *L. innocua* HPB13. Similarly, Strompfová et al. (2008) found the presence of a combination of the three structural genes *EntA, EntB*, and *EntP* in various food matrices including French and Slovak meat products. Other results are also in agreement with our findings showing the presence of the combination of these three enterocin genes in *E. faecium* strains from food and clinical sources (De Vuyst et al., 2003; Ogaki et al., 2016).

**Table 7:** Analysis for enterocins A, B, and P genes and antibacterial activity.

	MZF1	MZF2	MZF3	MZF4	MZF5
**Bacteriocin genes**					
Enterocin A (*EntA*)	+	+	-	+	+
Enterocin B (*EntB*)	+	+	+	+	+
Enterocin P (*EntP*)	+	+	-	+	+
**Antibacterial activity**			
*Listeria innocua* HPB13	+++	+++	+	+++	+++
*Enterococcus faecalis* ATCC 29212	+++	+	-	++	+++
*Staphylococcus aureus* ATCC 25923	-	-	-	-	-
*Escherichia coli* DH5α	-	-	-	-	-
*Pseudomonas aeruginosa* PAO1	-	-	-	-	-
*Salmonella typhimurium* ATCC 14028	-	-	-	-	-


*-, Absence of activity; +, weak activity; ++, moderate activity; +++, high activity.*

However, all the five *E. faecium* isolates displayed no inhibition of the reference strain *S. aureus* ATCC 25923, neither against the Gram-negative indicator bacteria used in this study. Besides, they showed inhibitory potential, strain-dependent, against *E. faecalis* ATCC 29212 except for MZF3 that showed no activity against this indicator strain. All the results of antibacterial activity are summarized in **Table 7**.

In our investigation, preliminary treatments on culture-free supernatant (CFS) have been applied in purpose to determine the nature of the antimicrobial compound. The activity of the inhibitory substance was retained in the presence of catalase indicating that the microbial inhibition is not due to H_2_O_2_, but not in the presence of proteinase K, reflecting the proteinaceous nature of the antimicrobial substance, also known as bacteriocin which is in our case an enterocin.

## Conclusion

To our knowledge, this is the first report on the study of *E. faecium* isolated from a Tunisian artisanal biotope called “Dried Ossban.” The outcomes of this work claim that enterococcal isolates are resistant against the harsh gastrointestinal conditions (acidity and bile salts). The safety assessment revealed that these bacteria were susceptible to clinically relevant antibiotics such as vancomycin. They do not have the capacity to produce histamine, the more toxic biogenic amine, and most of the *E. faecium* strains do not harbor the virulence genes for *agg, gelE, esp*, and *ace*. Conversely, all the isolates possess at least one enterocin and could efficiently inhibit the growth of *Listeria* spp. These results showed that these strains, in particular MZF5, according to its high adhesion capacity, absence of cytotoxicity and virulence genes, may be considered as interesting candidates for future use as probiotics and bioprotective cultures for application in the food and/or feed industries. Further tests will be needed to evaluate their technological characteristics and behavior in meat products.

## Author Contributions

MZ carried out most of the experimental work, interpreted the data, and wrote the manuscript. MC and OM contributed to the PCR assays. MB helped with the identification of bacteria by MALDI-TOF MS. MOF and NC designed and supervised the entire project. All authors edited the manuscript, read and approved the final version of the manuscript.

## Conflict of Interest Statement

The authors declare that the research was conducted in the absence of any commercial or financial relationships that could be construed as a potential conflict of interest.
